# Qualitative Controller Synthesis for Consumption Markov Decision Processes

**DOI:** 10.1007/978-3-030-53291-8_22

**Published:** 2020-06-16

**Authors:** František Blahoudek, Tomáš Brázdil, Petr Novotný, Melkior Ornik, Pranay Thangeda, Ufuk Topcu

**Affiliations:** 8 Microsoft Research Lab, Redmond, WA USA; 9 University of Southern California, Los Angeles, CA USA; 10 The University of Texas at Austin, Austin, USA; 11 Masaryk University, Brno, Czech Republic; 12 University of Illinois at Urbana-Champaign, Urbana, USA

## Abstract

Consumption Markov Decision Processes (CMDPs) are probabilistic decision-making models of resource-constrained systems. In a CMDP, the controller possesses a certain amount of a critical resource, such as electric power. Each action of the controller can consume some amount of the resource. Resource replenishment is only possible in special *reload states,* in which the resource level can be reloaded up to the full capacity of the system. The task of the controller is to prevent resource exhaustion, i.e. ensure that the available amount of the resource stays non-negative, while ensuring an additional linear-time property. We study the complexity of strategy synthesis in consumption MDPs with almost-sure Büchi objectives. We show that the problem can be solved in polynomial time. We implement our algorithm and show that it can efficiently solve CMDPs modelling real-world scenarios.

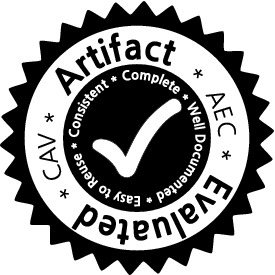



## Introduction

In the context of formal methods, controller synthesis typically boils down to computing a strategy in an *agent-environment* model, a nondeterministic state-transition model where some of the nondeterministic choices are resolved by the controller and some by an uncontrollable environment. Such models are typically either two-player graph games with an adversarial environment or Markov decision process (MDPs); the latter case being apt for modelling statistically predictable environments. In this paper, we consider controller synthesis for *resource-constrained MDPs*, where the computed controller must ensure, in addition to satisfying some linear-time property, that the system’s operation is not compromised by a lack of necessary resources.

*Resource-Constrained Probabilistic Systems.* *Resource-constrained* systems need a supply of some resource (e.g. power) for steady operation: the interruption of the supply can lead to undesirable consequences and has to be avoided. For instance, an autonomous system, e.g. an autonomous electric vehicle (*AEV*), is not able to draw power directly from an endless source. Instead, it has to rely on an internal storage of the resource, e.g. a battery, which has to be replenished in regular intervals to prevent resource exhaustion. Practical examples of AEVs include driverless cars, drones, or planetary rovers [8]. In these domains, resource failures may cause a costly mission failure and even safety risks. Moreover, the operation of autonomous systems is subject to probabilistic uncertainty [54]. Hence, in this paper, we study the resource-constrained strategy synthesis problem for MDPs.

*Models of Resource-Constrained Systems & Limitations of Current Approaches.* There is a substantial body of work in the area of verification of resource-constrained systems [3, 5, 7, 9, 11, 23, 38, 39, 53, 58]. The typical approach is to model them as finite-state systems augmented with an integer-valued counter representing the current *resource level,* i.e. the amount of the resource present in the internal storage. The resource constraint requires that the resource level never drops below zero.^1^ In the well-known *energy* model [11, 23], each transition is labelled by an integer, and performing an $\ell$-labelled transition results in $\ell$ being added to the counter. Thus, negative numbers stand for resource consumption while positive ones represent re-charging by the respective amount. Many variants of both MDP and game-based energy models were studied, as detailed in the related work. In particular, [26] considers controller synthesis for energy MDPs with qualitative Büchi and parity objectives. The main limitation of energy-based agent-environment models is that in general, they are not known to admit polynomial-time controller synthesis algorithms. Indeed, already the simplest problem, deciding whether a non-negative energy can be maintained in a two-player energy game, is at least as hard as solving mean-payoff graph games [11]; the complexity of the latter being a well-known open problem [45]. This hardness translates also to MDPs [26], making polynomial-time controller synthesis for energy MDPs impossible without a theoretical breakthrough.

*Consumption models,* introduced in [14], offer an alternative to energy models. In a consumption model, a non-negative integer, $cap$, represents the maximal amount of the resource the system can hold, e.g. the battery capacity. Each transition is labelled by a non-negative number representing the amount of the resource *consumed* when taking the transition (i.e., taking an $\ell$-labelled transition decreases the resource level by $\ell$). The resource replenishment is different from the energy approach. The consumption approach relies on the fact that reloads are often *atomic events*, e.g. an AEV plugging into a charging station and waiting to finish the charging cycle. Hence, some states in the consumption model are designated as *reload states,* and whenever the system visits a reload state, the resource level is replenished to the full capacity $cap$. Modelling reloads as atomic events is natural and even advantageous: consumption models typically admit more efficient analysis than energy models [14, 47]. However, consumption models have not yet been considered in the probabilistic setting.

*Our Contribution.* We study strategy synthesis in consumption MDPs with Büchi objectives. Our main theoretical result is stated in the following theorem.

### Theorem 1

Given a consumption MDP $\mathcal {M}$ with a capacity $cap$, an initial resource level $0\le d \le cap$, and a set *T* of accepting states, we can decide, in polynomial time, whether there exists a strategy $\sigma$ such that when playing according to $\sigma$, the following *consumption-Büchi objectives* are satisfied:

- Starting with resource level *d*, the resource level never^2^ drops below 0.
- With probability 1, the system visits some state in *T* infinitely often.

Moreover, if such a strategy exists then we can compute, in polynomial time, its polynomial-size representation.

For the sake of clarity, we restrict to proving Theorem 1 for a natural sub-class of MDPs called *decreasing consumption MDPs,* where there are no cycles of zero consumption. The restriction is natural (since in typical resource-constrained systems, each action – even idling – consumes some energy, so zero cycles are unlikely) and greatly simplifies presentation. In addition to the theoretical analysis, we implemented the algorithm behind Theorem 1 and evaluated it on several benchmarks, including a realistic model of an AEV navigating the streets of Manhattan. The experiments show that our algorithm is able to efficiently solve large CMDPs, offering a good scalability.

*Significance.* Some comments on Theorem 1 are in order. First, all the numbers in the MDP, and in particular the capacity $cap$, are encoded in binary. Hence, “polynomial time” means time polynomial in the encoding size of the MDP itself and in $\log ( cap )$. In particular, a naive “unfolding” of the MDP, i.e. encoding the resource levels between 0 and $cap$ into the states, does not yield a polynomial-time algorithm, but an exponential-time one, since the unfolded MDP has size proportional to $cap$. We employ a value-iteration-like algorithm to compute minimal energy levels with which one can achieve the consumption-Büchi objectives.

A similar concern applies to the “polynomial-size representation” of the strategy $\sigma$. To satisfy a consumption-Büchi objective, $\sigma$ generally needs to keep track of the current resource level. Hence, under the standard notion of a finite-memory (FM) strategy (which views FM strategies as transducers), $\sigma$ would require memory proportional to $cap$, i.e. a memory exponentially large w.r.t. size of the input. However, we show that for each state *s* we can partition the integer interval $[0,\ldots , cap ]$ into polynomially many sub-intervals $I_1^s,\ldots ,I_k^s$ such that, for each $1\le j \le k$, the strategy $\sigma$ picks the same action whenever the current state is *s* and the current resource level is in $I_j^s$. As such, the endpoints of the intervals are the only extra knowledge required to represent $\sigma$, a representation which we call a *counter selector*. We instrument our main algorithm so as to compute, in polynomial time, a polynomial-size counter selector representing the witness strategy $\sigma$.

Finally, we consider linear-time properties encoded by Büchi objectives over the states of the MDP. In essence, we assume that the translation of the specification to the Büchi automaton and its product with the original MDP model of the system were already performed. Probabilistic analysis typically requires the use of deterministic Büchi automata, which cannot express all linear-time properties. However, in this paper we consider qualitative analysis, which can be performed using restricted versions of non-deterministic Büchi automata that are still powerful enough to express all $\omega$-regular languages. Examples of such automata are limit-deterministic Büchi automata [51] or good-for-MDPs automata [41]. Alternatively, consumption MDPs with parity objectives could be reduced to consumption-Büchi MPDs using the standard parity-to-Büchi MDP construction [25, 30, 32, 33]. We abstract from these aspects and focus on the technical core of our problem, solving consumption-Büchi MDPs.

Consequently, to our best knowledge, we present the first polynomial-time algorithm for controller synthesis in resource-constrained MDPs with $\omega$-regular objectives.

*Related Work.* There is an enormous body of work on energy models. Stemming from the models introduced in [11, 23], the subsequent work covered energy games with various combinations of objectives [10, 12, 13, 18, 20, 21, 27, 48], energy games with multiple resource types [15, 24, 28, 31, 37, 43, 44, 57] or the variants of the above in the MDP [17, 49], infinite-state [1], or partially observable [34] settings. As argued previously, the controller synthesis within these models is at least as hard as solving mean-payoff games. The paper [29] presents polynomial-time algorithms for non-stochastic energy games with special weight structures. Recently, an abstract algebraic perspective on energy models was presented in [22, 35, 36].

Consumption systems were introduced in [14] in the form of consumption games with multiple resource types. Minimizing mean-payoff in automata with consumption constraints was studied in [16].

Our main result requires, as a technical sub-component, solving the *resource-safety* (or just *safety*) problem in consumption MDPs, i.e. computing a strategy which prevents resource exhaustion. The solution to this problem consists (in principle) of a Turing reduction to the problem of minimum cost reachability in two-player games with non-negative costs. The latter problem was studied in [46], with an extension to arbitrary costs considered in [19] (see also [40]). We present our own, conceptually simple, value-iteration-like algorithm for the problem, which is also used in our implementation.

Elements of resource-constrained optimization and minimum-cost reachability are also present in the line of work concerning *energy-utility quantiles* in MDPs [4–7, 42]. In this setting, there is no reloading in the consumption- or energy-model sense, and the task is typically to minimize the total amount of the resource consumed while maximizing the probability that some other objective is satisfied.

*Paper Organization & Outline of Techniques.* After the preliminaries (Sect. 2), we present counter selectors in Sect. 3. The next three sections contain the three main steps of our analysis. In Sect. 4, we solve the safety problem in consumption MDPs. The technical core of our approach is presented in Sect. 5, where we solve the problem of *safe positive reachability*: finding a resource-safe strategy which ensures that the set *T* of accepting states is visited with positive probability. Solving consumption-Büchi MDPs then, in principle, consists of repeatedly applying a strategy for safe positive reachability of *T*, ensuring that the strategy is “re-started” whenever the attempt to reach *T* fails. Details are given in Sect. 6. Finally, Sect. 7 presents our experiments. Due to space constraints, most technical proofs were moved to the full version.

## Preliminaries

We denote by $\mathbb {N}$ the set of all non-negative integers and by $\overline{\mathbb {N}}$ the set $\mathbb {N}\cup \{\infty \}$. Given a set *I* and a vector $\mathbf {v}\in \overline{\mathbb {N}}^{I}$ of integers indexed by *I*, we use $\mathbf {v}(i)$ to denote the *i*-component of $\mathbf {v}$. We assume familiarity with basic notions of probability theory. In particular, a *probability distribution* on an at most countable set *X* is a function $f:X \rightarrow [0,1]$ s.t. $\sum _{x\in X} f(x) = 1$. We use $\mathcal {D}(X)$ to denote the set of all probability distributions on *X*.

### Definition 1

**(CMDP).** A *consumption Markov decision process* (CMDP) is a tuple $\mathcal {M}= (S, A, \varDelta , C, R , cap )$ where $S$ is a finite set of *states*, $A$ is a finite set of *actions*, $\varDelta :S\times A\rightarrow \mathcal {D}(S)$ is a total *transition function*, $C:S\times A\rightarrow \mathbb {N}$ is a total *consumption function*, $R \subseteq S$ is a set of *reload states* where the resource can be reloaded, and $cap$ is a *resource capacity*.

**Fig. 1.**
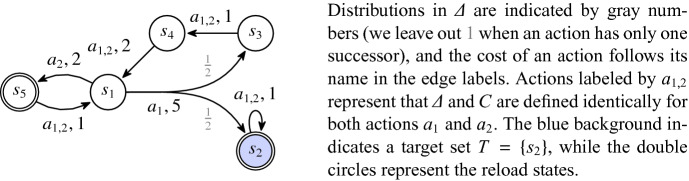
CMDP $\mathcal {M}=(\{s_1, s_2, s_3, s_4, s_5\}, \{a_1, a_2\}, \varDelta , C, \{s_2, s_5\}, 20)$. Details are given on the right.

Figure 1 shows a visual representation of an CMDP. We denote by $$\mathcal {M}(R')$$ for $$ R '\subseteq S$$ the CMDP obtained from $$\mathcal {M}$$ by changing the set of reloads to $$R'$$. For $$s\in S$$ and $$a\in A$$, we denote by $$ Succ (s,a)$$ the set $$\{t\mid \varDelta (s,a)(t)>0\}$$. A *path* is a (finite or infinite) state-action sequence $$\alpha =s_1a_1s_2a_2s_3\dots \in (S\times A)^\omega \cup (S\cdot A)^*\cdot S$$ such that $$s_{i+1}\in Succ (s_i,a_i)$$ for all *i*. We define $$\alpha _{i}=s_i$$ and $$ Act ^{i}(\alpha )=a_i$$. We use $$\alpha _{..i}$$ for the finite prefix $$s_1 a_1 \dots s_i$$ of $$\alpha $$, $$ \alpha _{i..} $$ for the suffix $$ s_i a_i\dots $$, and $$\alpha _{i..j}$$ for the infix $$s_ia_i\ldots s_j$$. A finite path is a *cycle* if it starts and ends in the same state and is *simple* if none of its infixes forms a cycle. The *length* of a path $$\alpha $$ is the number $$len(\alpha )$$ of actions on $$\alpha $$ and $$len(\alpha )=\infty $$ if $$\alpha $$ is infinite.

A CMDP is *decreasing* if for every cycle $s_1 a_1s_2 \ldots a_{k-1}s_k$ there exists $1 \le i < k$ such that $C(s_i,a_i) >0$. Throughout this paper we consider only decreasing CMDPs. The only place where this assumption is used are the proofs of Theorem 4 and Theorem 8.

An infinite path is called a *run*. We typically name runs by variants of the symbol $\varrho$. The set of all runs in $\mathcal {M}$ is denoted  . A finite path is called *history*. The set of all possible histories of $\mathcal {M}$ is  or simply . We use $last(\alpha )$ for the last state of $\alpha$. Let $\alpha$ be a history with $last(\alpha ) =s_1$ and $\beta =s_1a_1s_2a_2\ldots$; we define a *joint path* as $\alpha \odot \beta = \alpha a_1s_2a_2\ldots$.

A *strategy* for $\mathcal {M}$ is a function  assigning to each history an action to play. A strategy is *memoryless* if $\sigma (\alpha ) = \sigma (\beta )$ whenever $last(\alpha ) = last(\beta )$. We do not consider randomized strategies in this paper, as they are non-necessary for qualitative $\omega$-regular objectives on finite MDPs [30, 32, 33].

A computation of $\mathcal {M}$ under the control of a given strategy $\sigma$ from some initial state $s\in S$ creates a path. The path starts with $s_1=s$. Assume that the current path is $\alpha$ and let $s_i= last(\alpha )$ (we say that $\mathcal {M}$ is currently in $s_i$). Then the next action on the path is $a_{i}=\sigma (\alpha )$ and the next state $s_{i+1}$ is chosen randomly according to $\varDelta (s_i, a_{i})$. Repeating this process *ad infinitum* yields an infinite sample run $\varrho$. We say that $\varrho$ is $\sigma$*-compatible* if it can be produced using this process, and *s-initiated* if it starts in *s*. We denote the set of all $\sigma$-compatible, *s*-initiated runs by .

We denote by $\mathbb {P}^{\sigma }_{\mathcal {M},s}(\mathsf {A})$ the probability that a sample run from  belongs to a given measurable set of runs $\mathsf {A}$. For details on the formal construction of measurable sets of runs as well as the probability measure $\mathbb {P}^{\sigma }_{\mathcal {M},s}$ see [2]. Throughout the paper, we drop the $_\mathcal {M}$ subscripts in symbols whenever $\mathcal {M}$ is known from the context.

### Resource: Consumption, Levels, and Objectives

We denote by $cap (\mathcal {M})$ the battery capacity in the MDP $\mathcal {M}$. A resource is consumed along paths and can be reloaded in the reload states up to the full capacity. For a path $\alpha =s_1a_1s_2\ldots$ we define the consumption of $\alpha$ as $cons (\alpha ) = \sum _{i=1}^{len(\alpha )}C(s_i, a_i)$ (since the consumption is non-negative, the sum is always well defined, though possibly diverging). Note that $cons$ does not consider reload states at all. To accurately track the remaining amount of the resource, we use the concept of a *resource level*.

#### Definition 2

**(Resource level).** Let $\mathcal {M}$ be a CMDP with a set of reload states $R$, let $\alpha$ be a history, and let $0\le d \le cap (\mathcal {M})$ be an integer called *initial load*. Then the *energy level after* $\alpha$ *initialized by* *d*, denoted by $RL _{d}^{\mathcal {M}}(\alpha )$ or simply as $RL _{d}^{}(\alpha )$, is defined inductively as follows: for a zero-length history *s* we have $RL _{d}^{\mathcal {M}}(s) = d$. For a non-zero-length history $\alpha = \beta a t$ we denote $c = C({ last(\beta ) },{a})$, and put

$$ RL _{d}^{\mathcal {M}}(\alpha ) = {\left\{ \begin{array}{ll} RL _{d}^{\mathcal {M}}(\beta ) - c &{} \text {if } last(\beta ) \not \in R \text { and } c \le RL _{d}^{\mathcal {M}}(\beta ) \ne \bot \\ cap (\mathcal {M}) - c &{} \text {if } last(\beta ) \in R \text { and } c\le cap (\mathcal {M}) \text { and } RL _{d}^{\mathcal {M}}(\beta ) \ne \bot \\ \bot &{} \text {otherwise} \end{array}\right. } $$

Consider $\mathcal {M}$ from Fig. 1 and the history $\alpha (i) = (s_1a_2s_5a_2)^is_1$ with *i* as a parameter. We have $cons (\alpha (i)) = 3i$ and at the same time, following the inductive definition of $RL _{d}^{}(\alpha (i))$ we have $RL _{2}^{}(\alpha (i)) = 19$ for all $i \ge 1$ as the resource is reloaded every time in $s_5$. This generalizes into the following. Let $\alpha$ be a history and let $f,l \ge 0$ be the minimal and maximal indices *i* such that $\alpha _{i}\in R$, respectively. For $RL _{d}^{}(\alpha ) \ne \bot$, it holds $RL _{d}^{}(\alpha _{..i}) = d - cons (\alpha _{..i})$ for all $i \le f$ and $RL _{d}^{}(\alpha ) = cap (\mathcal {M}) - cons (\alpha _{l..})$. Further, for each history $\alpha$ and *d* such that $e= RL _{d}^{}(\alpha )\ne \bot$, and each history $\beta$ suitable for joining with $\alpha$ it holds that $RL _{d}^{}(\alpha \odot \beta ) = RL _{e}^{}(\beta )$.

A run $\varrho$ is *d-safe* if and only if the energy level initialized by *d* is a non-negative number for each finite prefix of $\rho$, i.e. if for all $i > 0$ we have $RL _{d}^{}( \varrho _{..i}) \ne \bot$. We say that a run is safe if it is $cap (\mathcal {M})$-safe. The next lemma follows immediately from the definition of an energy level.

#### Lemma 1

Let $\varrho =s_1a_1s_2\ldots$ be a *d*-safe run for some *d* and let $\alpha$ be a history such that $last(\alpha ) =s_1$. Then the run $\alpha \odot \varrho$ is *e*-safe if $RL _{e}^{}(\alpha )\ge d$.

#### Example 1

Recall the CMDP and the parameterized history $\alpha (i)$ from above. We know that $RL _{2}^{}(\alpha (i)) = 19$ for all *i*. Therefore, a strategy that always picks $a_2$ in $s_1$ is *d*-safe in $s_1$ for all $d \ge 2$. On the other hand, a strategy that always picks $a_1$ in $s_1$ is *not* *d*-safe in $s_1$ for any $0\le d \le 20 = cap (\mathcal {M})$ because for all runs $\varrho$ that visit $s_3$ at least three times before $s_2$ we have $RL _{d}^{}( \varrho ) = \bot$.

*Objectives.* An *objective* is a set of runs. The objective $\mathsf {SafeRuns}(d)$ contains exactly *d*-safe runs. Given a *target set* $T\subseteq S$ and $i \in \mathbb {N}$, we define  to be the set of all runs that reach some state from $T$ within the first *i* steps. We put $\mathsf {Reach}_{T}= \bigcup _{i \in \mathbb {N}} \mathsf {Reach}_{T}^i$. Finally, the set .

*Problems.* We solve three main qualitative problems for CMDPs, namely *safety*, *positive reachability*, and *Büchi*.

Let us fix a state *s* and a target set of states $$ T$$. We say that a strategy $$\sigma $$ is *d-safe in* *s* if . We say that $$\sigma $$ is $$T$$*-positive* *d-safe in* *s* if it is *d*-safe in *s* and $$\mathbb {P}^{\sigma }_{s}(\mathsf {Reach}_{T}) > 0$$, which means that there exists a run in  that visits $$T$$. Finally, we say that $$\sigma $$ is $$T$$*-Büchi* *d-safe in a state* *s* if it is *d*-safe in *s* and $$ \mathbb {P}^{\sigma }_{s}(\mathsf {B\ddot{u}chi}_{T}) = 1$$.

The vectors $$ Safe $$, $$ SafePR _{T}$$ (PR for “positive reachability”), and $$ SafeB\ddot{u}chi _{T}$$ of type $$\overline{\mathbb {N}}^S$$ contain, for each $$s\in S$$, the minimal *d* such that there exists a strategy that is *d*-safe in *s*, $$T$$-positive *d*-safe in *s*, and $$T$$-Büchi *d*-safe in *s*, respectively, and $$\infty $$ if no such strategy exists.

The problems we consider for a given CMDP are:

- *Safety:* compute the vector $Safe$ and a strategy that is $Safe (s)$-safe in every $s \in S$.
- *Positive reachability:* compute the vector $$ SafePR _{T}$$ and a strategy that is $$ T$$-positive $$ SafePR _{T}(s) $$-safe in every state *s*.
- *Büchi:* compute $$ SafeB\ddot{u}chi _{T}$$ and a strategy that is $$ T$$-Büchi $$ SafeB\ddot{u}chi _{T}(s) $$-safe in every state *s*.

#### Example 2

Now consider again the *d*-safe strategy from Example 1 that always picks $a_2$; such a strategy is 2-safe in $s_1$, but is not useful if we attempt to eventually reach $T$. Hence memoryless strategies are not sufficient in our setting. Consider, instead, a strategy $\sigma$ that picks $a_1$ in $s_1$ whenever the current resource level is at least 10 and picks $a_2$ otherwise. Such a strategy is 2-safe in $s_1$ and guarantees reaching $s_2$ with a positive probability: we need at least 10 units of energy to return to $s_5$ in the case we are unlucky and picking $a_1$ leads us to $s_3$. If we are lucky, $a_1$ leads us to $s_2$ by consuming just 5 units of the resource, witnessing that $\sigma$ is $T$-positive. As a matter of fact, during *every* revisit of $s_5$ there is a $\frac{1}{2}$ chance of hitting $s_2$ during the next try, so $\sigma$ actually ensures that $s_2$ is visited with probability 1.

Solving a CMDP is substantially different from solving a consumption 2-player game [14]. Indeed, imagine that in $\mathcal {M}$ from Fig. 1, the outcome of the action $a_1$ from state $s_1$ is resolved by an adversarial player. In such a game, the strategy $\sigma$ does not produce any run that reaches $s_2$. In fact, there would be no strategy that guarantees reaching $T$ in a 2-player game like this at all.

The strategy $\sigma$ from our example uses finite memory to track the resource level exactly. We describe an efficient representation of such strategies in the next section.

## Counter Strategies

In this section, we define a succinct representation of finite-memory strategies via so called counter selectors. Under the standard definition, a strategy $\sigma$ is a *finite memory* strategy, if $\sigma$ can be encoded by a *memory structure*, a type of finite transducer. Formally, a memory structure is a tuple $\mu =(M, nxt , up ,m_0)$ where $M$ is a finite set of *memory elements*, $nxt :M\times S\rightarrow A$ is a *next action* function, $up :M\times S\times A\times S\rightarrow M$ is a *memory update* function, and $m_0:S\rightarrow M$ is the *memory initialization function*. The function $up$ can be lifted to a function  as follows.

$$ up ^*(m,\alpha ) = {\left\{ \begin{array}{ll} m &{} \text {if } \alpha = s \text { has length 0}\\ up \big ( up ^*(m,\beta ), last(\beta ) , a, t\big ) &{} \text {if } \alpha = \beta a t \text { for some } a\in A\text { and } t\in S\\ \end{array}\right. } $$

The structure $\mu$ encodes a strategy $\sigma _\mu$ such that for each history $\alpha =s_1a_1s_2\ldots s_n$ we have $\sigma _\mu (\alpha ) = nxt \big ( up ^*(m_0(s_1),\alpha ), s_n\big )$.

In our setting, strategies need to track energy levels of histories. Let us fix an CMDP $\mathcal {M}= (S, A, \varDelta , C, R , cap )$. A non-exhausted energy level is always a number between 0 and $cap (\mathcal {M})$, which can be represented with a binary-encoded bounded counter. We call strategies with such counters *finite counter (FC) strategies*. An FC strategy selects actions to play according to *selection rules*.

### Definition 3

**(Selection rule).** A *selection rule* $\varphi$ for $\mathcal {M}$ is a partial function from the set $\{0,\ldots , cap (\mathcal {M})\}$ to *A*. Undefined value for some *n* is indicated by $\varphi (n)=\bot$.

We use $$ dom (\varphi ) = \{n \in \{0 ,\ldots , cap (\mathcal {M})\} \mid \varphi (n)\ne \bot \}$$ to denote the domain of $$\varphi $$ and we use  or simply  for the set of all selection rules for $$\mathcal {M}$$. Intuitively, a selection according to rule $$\varphi $$ selects the action that corresponds to the largest value from $$ dom (\varphi )$$ that is not larger than the current energy level. To be more precise, if $$ dom (\varphi ) $$ consists of numbers $$ n_1< n_2< \cdots < n_k $$, then the action to be selected in a given moment is $$\varphi (n_i)$$, where $$n_i$$ is the largest element of $$ dom (\varphi ) $$ which is less then or equal to the current amount of the resource. In other words, $$\varphi (n_i)$$ is to be selected if the current resource level is in $$ [n_i,n_{i+1}) $$ (putting $$ n_{k+1} = \infty $$).

### Definition 4

**(Counter selector).** A *counter selector* for $\mathcal {M}$ is a function .

A counter selector itself is not enough to describe a strategy. A strategy needs to keep track of the energy level throughout the path. With a vector $$\mathbf {r}\in \{0,\ldots , cap (\mathcal {M})\}^{S}$$ of initial resource levels, each counter selector $$\varSigma $$ defines a strategy $$\varSigma ^\mathbf {r}$$ that is encoded by the following memory structure $$ (M, nxt , up ,m_0)$$ with $$a\in A$$ being a globally fixed action (for uniqueness). We stipulate that $$\bot < n$$ for all $$n\in \mathbb {N}$$.

- $$M= \{\bot \} \cup \{0,\ldots , cap (\mathcal {M})\}$$.
- Let $$m\in M$$ be a memory element, let $$s\in S$$ be a state, let $$n\in dom (\varSigma (s))$$ be the largest element of $$ dom (\varSigma (s))$$ such that $$n \le m$$. Then $$ nxt (m,s) = \varSigma (s)(n)$$ if *n* exists, and $$ nxt =a$$ otherwise.
- The function $up$ is defined for each $m\in M, a\in A, s,t\in S$ as follows.
  $$ up (m,s,a,t) = {\left\{ \begin{array}{ll} m - C(s,a) &{} \text {if } s \not \in R \text { and } C(s,a) \le m \ne \bot \\ cap (\mathcal {M}) -C(s,a) &{} \text {if } s \in R \text { and } C(s,a) \le cap (\mathcal {M}) \text { and } m \ne \bot \\ \bot &{} \text {otherwise}. \end{array}\right. } $$
- The function $$ m_0$$ is $$ m_0(s) = \mathbf {r}(s)$$.

A strategy $\sigma$ is a finite counter (FC) strategy if there is a counter selector $\varSigma$ and a vector $\mathbf {r}$ such that $\sigma = \varSigma ^{\mathbf {r}}$. The counter selector can be imagined as a finite-state device that implements $\sigma$ using $\mathcal {O}(\log ( cap (\mathcal {M})))$ bits of additional memory (counter) used to represent numbers $0,1,\ldots , cap (\mathcal {M})$. The device uses the counter to keep track of the current resource level, the element $\bot$ representing energy exhaustion. Note that a counter selector can be exponentially more succinct than the corresponding memory structure.

### Example 3

Consider again the CMDP $\mathcal {M}$ in Fig. 1 and a counter selector $\varSigma$ defined as follows: Let $\varphi$ be a selection rule with $dom (\varphi ) = \{0,10\}$ such that $\varphi (0) = a_2$ and $\varphi (10) = a_1$. Then let $\varphi '$ be a selection rule such that $dom (\varphi ')=\{0\}$ and $\varphi (0)=a_1$. Finally, let $\varSigma$ be a counter selector such that $\varSigma (s_1) = \varphi$ and $\varSigma (s_i) = \varphi '$ for all $i\ne 1$. Then, for a vector of initial resource levels $\mathbf {r}$, the strategy $\sigma$ informally described in Example 2 can be formally represented by putting $\sigma = \varSigma ^{\mathbf {r}}$. Note that for any $\mathbf {r}$ with $\mathbf {r}(s_1)\ge 2$, $\mathbf {r}(s_2)\ge 0$, $\mathbf {r}(s_3)\ge 5$, $\mathbf {r}(s_4)\ge 4$, and $\mathbf {r}(s_5)\ge 0$ and for any state *s* of $\mathcal {M}$ the strategy $\varSigma ^{\mathbf {r}}$ is $\mathbf {r}(s)$-safe in *s*.

## Safety

In this section, we present an algorithm that computes, for each state, the minimal value *d* (if it exists) such that there exists a *d*-safe strategy from that state. We also provide the corresponding strategy. In the remainder of the section we fix an MDP $\mathcal {M}$.

A *d*-safe run has the following two properties: (i) It consumes at most *d* units of the resource (energy) before it reaches the first reload state, and (ii) it never consumes more than $$ cap (\mathcal {M})$$ units of the resource between 2 visits of reload states. To ensure (ii), we need to identify a maximal subset $$ R '\subseteq R $$ of reload states for which there is a strategy $$\sigma $$ that, starting in some $$r\in R '$$, can always reach $$ R '$$ again (within at least one step) using at most $$ cap (\mathcal {M})$$ resource units. The *d*-safe strategy we seek can be then assembled from $$\sigma $$ and from a strategy that suitably navigates towards $$ R '$$, which is needed for (i).

In the core of both properties (i) and (ii) lies the problem of *minimum cost reachability.* Hence, in the next subsection, we start with presenting necessary results on this problem.

### Minimum Cost Reachability

The problem of minimum cost reachability with non-negative costs was studied before [46]. Here we present a simple approach to the problem used in our implementation and most of the technical details are available in the full version.

#### Definition 5

Let $T\subseteq S$ be a set of *target* states, let $\alpha =s_1 a_1 s_2 \ldots$ be a finite or infinite path, and let $1 \le f$ be the smallest index such that $s_f\in T$. We define *consumption of* $\alpha$ *to* $T$ as $ReachCons _{\mathcal {M},T}(\alpha ) = cons (\alpha _{..f})$ if *f* exists and we set $ReachCons _{\mathcal {M},T}(\alpha ) = \infty$ otherwise. For a strategy $\sigma$ and a state $s\in S$ we define .

A *minimum cost reachability of* $T$ *from* *s* is a vector defined as

$$ MinReach _{\mathcal {M}, T}(s) = \inf \big \{ ReachCons _{\mathcal {M},T}(\sigma , s) \mid \sigma \text { is a strategy for }\mathcal {M}\big \}. $$

Intuitively, $$d= MinReach _{T}(s)$$ is the minimal initial load with which some strategy can ensure reaching *T* with consumption at most *d*, when starting in *s*. We say that a strategy $$\sigma $$ is optimal for $$ MinReach _{T}$$ if we have that $$ MinReach _{T}(s) = ReachCons _{T}(\sigma , s)$$ for all states $$s\in S$$.

We also define functions $ReachCons _{\mathcal {M}, T}^+$ and the vector $MinReach _{\mathcal {M},T}^+$ in a similar fashion with one exception: we require the index *f* from definition of $ReachCons _{\mathcal {M},T}(\alpha )$ to be strictly larger than 1, which enforces to take at least one step to reach *T*.

For the rest of this section, fix a target set *T* and consider the following functional $\mathcal {F}$:

$$ \mathcal {F}(\mathbf {v})(s) = {\left\{ \begin{array}{ll} \min \nolimits _{a\in A} \left( C(s,a) + \max \nolimits _{t\in Succ (s,a)} \mathbf {v}(t)\right) &{} s \not \in T\\ 0 &{} s \in T\end{array}\right. } $$

$\mathcal {F}$ is a simple generalization of the standard Bellman functional used for computing shortest paths in graphs. The proof of the following Theorem is rather standard and moved to the full version of the paper.

#### Theorem 2

Denote by *n* the length of the longest simple path in $$\mathcal {M}$$. Let $$\mathbf {x}_T$$ be a vector such that $$\mathbf {x}_T(s)=0$$ if $$s\in T$$ and $$\mathbf {x}_T(s)=\infty $$ otherwise. Then iterating $$\mathcal {F}$$ on $$\mathbf {x}_T$$ yields a fixpoint in at most *n* steps and this fixpoint equals $$ MinReach _{T}$$.

To compute $MinReach _{\mathcal {M}, T}^+$, we construct a new CMDP $\widetilde{\mathcal {M}}$ from $\mathcal {M}$ by adding a copy $\tilde{s}$ of each state $s \in S$ such that dynamics in $\tilde{s}$ is the same as in *s*; i.e. for each $a\in A$, $\varDelta (\tilde{s},a)=\varDelta (s,a)$ and $C(\tilde{s},a) = C(s,a)$. We denote the new state set as $\widetilde{S}$. We don’t change the set of reload states, so $\tilde{s}$ is *never* in *T*, even if *s* is. Given the new CMDP $\widetilde{\mathcal {M}}$ and the new state set as $\widetilde{S}$, the following lemma is straightforward.

#### Lemma 2

Let $\mathcal {M}$ be a CMDP and let $\widetilde{\mathcal {M}}$ be the CMDP constructed as above. Then for each state *s* of $\mathcal {M}$ it holds $MinReach _{\mathcal {M}, T}^+(s) = MinReach _{\widetilde{\mathcal {M}},T}(\tilde{s})$.

### Safely Reaching Reload States

In the following, we use $MinInitCons _\mathcal {M}$ (read *minimal initial consumption*) for the vector $MinReach _{\mathcal {M}, R }^+$ – minimal resource level that ensures we can surely reach a reload state in at least one step. By Lemma 2 and Theorem 2 we can construct $\widetilde{\mathcal {M}}$ and iterate the operator $\mathcal {F}$ for |*S*| steps to compute $MinInitCons _\mathcal {M}$. Note that *S* is the state space of $\mathcal {M}$ since introducing the new states into $\widetilde{\mathcal {M}}$ did not increase the length of the maximal simple path. However, we can avoid the construction of $\widetilde{\mathcal {M}}$ and still compute $MinInitCons _\mathcal {M}$ using a *truncated* version of the functional $\mathcal {F}$, which is the approach used in our implementation. We first introduce the following truncation operator:Then, we define a truncated functional $\mathcal {G}$ as follows:

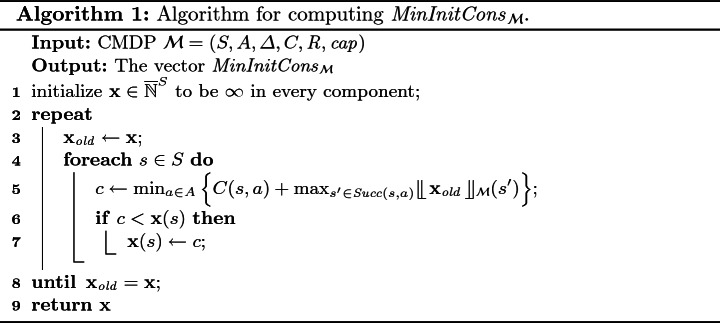


The following lemma connects the iteration of $\mathcal {G}$ on $\mathcal {M}$ with the iteration of $\mathcal {F}$ on $\widetilde{\mathcal {M}}$.

#### Lemma 3

Let $\varvec{\infty }\in \overline{\mathbb {N}}^{S}$ be a vectors with all components equal to $\infty$. Consider iterating $\mathcal {G}$ on $\varvec{\infty }$ in $\mathcal {M}$ and $\mathcal {F}$ on $\mathbf {x}_ R$ in $\widetilde{\mathcal {M}}$. Then for each $i\ge 0$ and each $s\in R$ we have $\mathcal {G}^i(\varvec{\infty })(s) = \mathcal {F}^i(\mathbf {x}_ R )(\tilde{s})$ and for every $s\in S\setminus R$ we have $\mathcal {G}^i(\varvec{\infty })(s) = \mathcal {F}^i(\mathbf {x}_ R )(s)$.

Algorithm 1 uses $\mathcal {G}$ to compute the vector $MinInitCons _\mathcal {M}.$

#### Theorem 3

Algorithm 1 correctly computes the vector $MinInitCons _\mathcal {M}$. Moreover, the repeat-loop terminates after at most $|S|$ iterations.

### Solving the Safety Problem

We want to identify a set $$ R '\subseteq R$$ such that we can reach $$ R '$$ in at least 1 step and with consumption at most $$ cap = cap (\mathcal {M})$$, from each $$r \in R '$$. This entails identifying the maximal $$ R '\subseteq R$$ such that $$ MinInitCons _{\mathcal {M}( R ')} \le cap $$ for each $$r\in R '$$. This can be done by initially setting $$ R '= R $$ and iteratively removing states that have $$ MinInitCons _{\mathcal {M}( R ')} > cap $$, from $$ R ' $$, as in Algorithm 2.
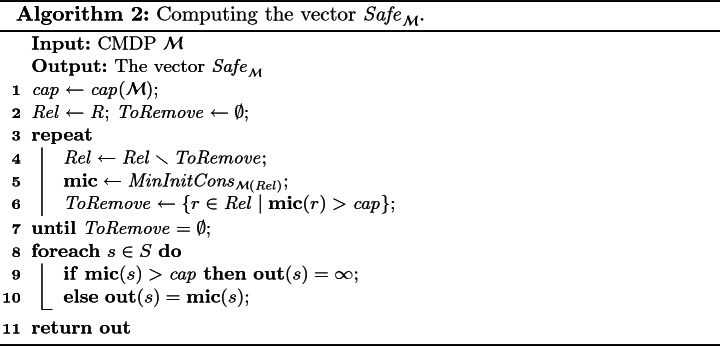


#### Theorem 4

Algorithm 2 computes the vector $Safe _{\mathcal {M}}$ in polynomial time.

#### Proof

The algorithm clearly terminates. Computing $MinInitCons _{\mathcal {M}( Rel )}$ on line 5 takes a polynomial number of steps per call due to Theorem 3 and since $\mathcal {M}( Rel )$ has asymptotically the same size as $\mathcal {M}$. Since the repeat loop performs at most $| R |$ iterations, the complexity follows.

As for correctness, we first prove that $$ {\mathbf {out}} \le Safe _\mathcal {M}$$. It suffices to prove for each $$s\in S$$ that upon termination, $$\mathbf {mic}(s) \le Safe _{\mathcal {M}}(s)$$ whenever the latter value is finite. Since $$ MinInitCons _{\mathcal {M}'}(s) \le Safe _{\mathcal {M}'}(s) $$ for each MDP $$ \mathcal {M}' $$ and each its state such that $$ Safe _{\mathcal {M}'}(s) <\infty $$, it suffices to show that $$ Safe _{\mathcal {M}( Rel )} \le Safe _{\mathcal {M}} $$ is an invariant of the algorithm (as a matter of fact, we prove that $$ Safe _{\mathcal {M}( Rel )} = Safe _{\mathcal {M}} $$). To this end, it suffices to show that at every point of execution $$ Safe _{\mathcal {M}}(t) = \infty $$ for each $$ t\in R \setminus Rel $$: indeed, if this holds, no strategy that is safe for some state $$s \ne t$$ can play an action *a* from *s* such that $$t \in Succ (s,a)$$, so declaring such states non-reloading does not influence the $$ Safe _{\mathcal {M}} $$-values. So denote by $$ Rel _i $$ the contents of $$ Rel $$ after the *i*-th iteration. We prove, by induction on *i*, that $$ Safe _{\mathcal {M}}(s) = \infty $$ for all $$ s \in R \setminus Rel $$. For $$ i = 0 $$ we have $$ R = Rel $$, so the statement holds. For $$ i>0 $$, let $$ s \in R \setminus Rel _{i}$$, and let $$ \sigma $$ be any strategy. If some run from  visits a state from $$ R \setminus Rel _{i-1} $$, then $$ \sigma $$ is not $$ cap $$-safe, by induction hypothesis. Now assume that all such runs only visit reload states from $$ Rel _{i-1}$$. Then, since $$ MinInitCons _{\mathcal {M}( Rel _{i-1})}(s)> cap $$, there must be a run  with $$ ReachCons _{ Rel _{i-1}}^+( \varrho )> cap $$. Assume that $$ \varrho $$ is $$ cap $$-safe in *s*. Since we consider only decreasing CMDPs, $$ \varrho $$ must infinitely often visit a reload state (as it cannot get stuck in a zero cycle). Hence, there exists an index $$ f>1 $$ such that $$ \varrho _{f}\in Rel _{i-1} $$, and for this *f* we have $$ RL _{ cap }^{}( \varrho _{..f}) = \bot $$, a contradiction. So again, $$\sigma $$ is not safe in *s*. Since there is no safe strategy from *s*, we have $$ Safe _{\mathcal {M}}(s) = \infty $$.

Finally, we need to prove that upon termination, $\mathbf {out} \ge Safe _{\mathcal {M}}$. Informally, per the definition of $\mathbf {out}$, from every state *s* we can ensure reaching a state of $Rel$ by consuming at most $\mathbf {out}(s)$ units of the resource. Once in $Rel$, we can ensure that we can again return to $Rel$ without consuming more than $cap$ units of the resource. Hence, when starting with $\mathbf {out}(s)$ units, we can surely prevent resource exhaustion.    $\square$

#### Definition 6

We call an action *a* *safe* in a state *s* if one of the following conditions holds:

- $$ s \not \in R $$ and $$ C(s,a) + \max _{t\in Succ (s,a)} { Safe _{\mathcal {M}}}(t) \le Safe _{\mathcal {M}}(s) $$; or
- $$ s \in R $$ and $$ C(s,a) + \max _{t\in Succ (s,a)} { Safe _{\mathcal {M}}}(t) \le cap (\mathcal {M}) $$.

Note that by the definition of $Safe _{\mathcal {M}} ,$ for each state *s* with $Safe _{\mathcal {M}}(s) < \infty$ there is always at least one action safe in *s*. For states *s* s.t. $Safe _{\mathcal {M}}(s) = \infty$, we stipulate all actions to be safe in *s*.

#### Theorem 5

Any strategy which always selects an action that is safe in the current state is $Safe _{\mathcal {M}}(s)$-safe in every state *s*. In particular, in each consumption MDP $\mathcal {M}$ there is a memoryless strategy $\sigma$ that is $Safe _{\mathcal {M}}(s)$-safe in every state *s*. Moreover, $\sigma$ can be computed in polynomial time.

#### Proof

The first part of the theorem follows directly from Definition 6, Definition 2 (resource levels), and from definition of *d*-safe runs. The second part is a corollary of Theorem 4 and the fact that in each state, the safe strategy from Definition 6 can fix one such action in each state and thus is memoryless. The complexity follows from Theorem 4.    $\square$

#### Example 4

Consider again the $\mathcal {M}$ from Fig. 1. *Algorithm* 1 returns, for input $\mathcal {M}$, the vector $\mathbf {mic} = (2, 1, 5, 4, 3)$. Algorithm 2 reuses $\mathbf {mic}$ on line 5 and returns it unchanged. Hence, the vector $\mathbf {mic}$ equals $Safe _\mathcal {M}$. The strategies described in Example 1 witness that $Safe (s_1) \le 2$. Here we see that there is no strategy that would be 1-safe in $s_1$.

## Positive Reachability

In this section, we focus on strategies that are safe and such that at least one run they produce visits a given set $T\subseteq S$ of *targets*. The main contribution of this section is Algorithm 3 used to compute such strategies as well as the vector $SafePR _{\mathcal {M},T}$ of minimal initial resource levels for which such a strategy exist. As before, for the rest of this section we fix a CMDP $\mathcal {M}$.

We define a function  ($SPR$ for safe positive reachability) s.t. for all $s\in S, a\in A$, and $\mathbf {x}\in \overline{\mathbb {N}}^S$ we haveThe $\max$ operator considers, for given *t*, the value $\mathbf {x}(t)$ and the values needed to survive from all possible outcomes of *a* other than *t*. Let  and *t* the outcome selected by $\min$. Intuitively, *v* is the minimal amount of resource needed to reach *t* with at least $\mathbf {x}(t)$ resource units, or survive if the outcome of *a* is different from *t*.

We now define a functional whose fixed point characterizes . We first define a two-sided version of the truncation operator from the previous section: the operator  such thatUsing the functions  and , we now define an auxiliary operator $$\mathcal {A}$$ and the main operator $$\mathcal {B}$$ as follows.Let $$ SafePR _{T}^i$$ be the vector such that for a state $$s\in S$$ the number $$d= SafePR _{T}^i(s)$$ is the minimal number $$ \ell $$ such that there exists a strategy that is $$\ell $$-safe in *s* and produces at least one run that visits $$T$$ within first *i* steps. Further, we denote by $$ \mathbf {y}_T$$ a vector such that

$$ \mathbf {y}_T(s) = {\left\{ \begin{array}{ll} Safe _{\mathcal {M}}(s) &{} \text {if } s \in T\\ \infty &{} \text {if } s \not \in T\end{array}\right. } $$

The following lemma can proved by a rather straightforward but technical induction.

### Lemma 4

Consider the iteration of $\mathcal {B}_{\mathcal {M}}$ on the initial vector $\mathbf {y}_T$. Then for each $i \ge 0$ it holds that $\mathcal {B}_{\mathcal {M}}^i(\mathbf {y}_T) = SafePR _{\mathcal {M},T}^i$.

The following lemma says that iterating $$ \mathcal {B}_{\mathcal {M}} $$ reaches a fixed point in a polynomial number of iterations. Intuitively, this is because when trying to reach $$ T$$, it doesn’t make sense to perform a cycle between two visits of a reload state (as this can only increase the resource consumption) and at the same time it doesn’t make sense to visit the same reload state twice (since the resource is reloaded to the full capacity upon each visit). The proof is straightforward and is omitted in the interest of brevity. Detailed proofs for Lemma 4 and Lemma 5 are available in the full version of the paper.

### Lemma 5

Let $K = | R | + (| R |+1)\cdot (|S|-| R |+1)$. Taking the same initial vector $\mathbf {y}_T$ as in Lemma 4, we have $\mathcal {B}_{\mathcal {M}}^{K}(\mathbf {y}_T) = SafePR _{\mathcal {M},T}$.

The computation of $SafePR _{\mathcal {M},T}$ and of the associated witness strategy is presented in Algorithm 3.
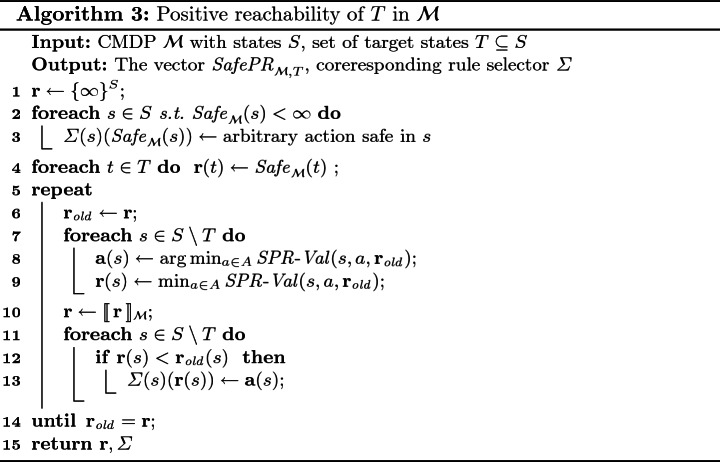


### Example 5

Consider again the CMDP $\mathcal {M}$ from Fig. 1. After one iteration of the loop on line 5, we have $\mathbf {r} = (10, 0, \infty , \infty , \infty )$, as $\mathbf {r}$ is only finite for $s_2$ before this iteration. In the next iteration, we have $\mathbf {r}=(10, 0, \infty , 12, 0)$. Thus, the next iteration changes the value for $s_1$ to 2 and in the end, we end up with $\mathbf {r} = (2, 0, 4, 5, 0)$. The iteration with $\mathbf {r}(s_1)=10$ influences the selector $\varSigma$. Note that the computed $\mathbf {r}$ and $\varSigma$ match those mentioned in Example 3.

### Theorem 6

The Algorithm 3 always terminates after a polynomial number of steps, and upon termination, $\mathbf {r} = SafePR _{\mathcal {M},T}$.

### Proof

The repeat loop on lines 1–4 initialize $\mathbf {r}$ to $\mathbf {y}_T$. The repeat loop on lines 5–14 then iterates the operator $\mathcal {B}$. By Lemma 5, the iteration reaches a fixed point in at most *K* steps, and this fixed point equals $SafePR _{\mathcal {M},T}$. The complexity bound follows easily, since *K* is of polynomial magnitude.

The most intricate part of our analysis is extracting a strategy that is $$ T$$-positive $$ SafePR _{\mathcal {M},T}(s) $$-safe in every state *s*.

### Theorem 7

Let $\mathbf {v} = SafePR _{\mathcal {M},T}$. Upon termination of Algorithm 3, the computed selector $\varSigma$ has the property that the finite counter strategy $\varSigma ^{\mathbf {v}}$ is, for each state $s\in S$, $T$-positive $\mathbf {v}(s)$-safe in *s*. That is, a polynomial-size finite counter strategy for the positive reachability problem can be computed in polynomial time.

The rest of this section is devoted to the proof of Theorem 7. The complexity follows from Theorem 6. Indeed, since the algorithm has a polynomial complexity, also the size of $\varSigma$ is polynomial. The correctness proof is based on the following invariant of the main repeat loop: the finite counter strategy $\pi = \varSigma ^{\mathbf {r}}$ has these properties:

- Strategy $\pi$ is $Safe _\mathcal {M}(s)$-safe in every state $s\in S$; in particular, we have for $l=\min \{\mathbf {r}(s), cap (\mathcal {M})\}$ that $RL _{l}^{}(\alpha ) \ne \bot$ for every finite path $\alpha$ produced by $\pi$ from *s*.
- For each state $s\in S$ such that $\mathbf {r}(s) \le cap (\mathcal {M})$ there exists a $\pi$-compatible finite path $\alpha =s_1a_1s_2\ldots s_n$ such that $s_1=s$ and $s_n\in T$ and such that “the resource level with initial load $\mathbf {r}(s)$ never decreases below $\mathbf {r}$ along $\alpha$”, which means that for each prefix $\alpha _{..i}$ of $\alpha$ it holds $RL _{\mathbf {r}(s)}^{}(\alpha _{..i})\ge \mathbf {r}(s_i)$.

The theorem then follows from this invariant (parts (a) and the first half of (b)) and from Theorem 6. We start with the following support invariant, which is easy to prove.

### Lemma 6

The inequality $\mathbf {r}\ge Safe _{\mathcal {M}}$ is an invariant of the main repeat-loop.

*Proving Part (a) of the Main Invariant.* We use the following auxiliary lemma.

### Lemma 7

Assume that $\varSigma$ is a counter selector such that for all $s \in S$ such that $Safe (s)<\infty$:

1. $$ Safe (s)\in dom (\varSigma (s)) $$.
2. For all $x \in dom (\varSigma (s))$, for $a = \varSigma (s)(x)$ and for all $t\in Succ (s,a)$ we have $RL _{x}^{}(sat) = d - C(s,a)\ge Safe (t)$ where $d=x$ for $s\notin R$ and $d= cap (\mathcal {M})$ otherwise.

Then for each vector $$ \mathbf {y} \ge Safe $$ the strategy $$ \pi =\varSigma ^{\mathbf {y}} $$ is $$ Safe (s)$$-safe in every state *s*.

### Proof

Let *s* be a state such that $\mathbf {y}(s) < \infty$. It suffices to prove that for every $\pi$-compatible finite path $\alpha$ started in *s* it holds $\bot \ne RL _{\mathbf {y}(s)}^{}(\alpha )$. We actually prove a stronger statement: $\bot \ne RL _{\mathbf {y}(s)}^{}(\alpha ) \ge Safe ( last(\alpha ) )$. We proceed by induction on the length of $\alpha$. If $len(\alpha )=0$ we have $RL _{\mathbf {y}(s)}^{}(\alpha ) = \mathbf {y}(s) \ge Safe _{\mathcal {M}}(s)\ge 0$. Now let $\alpha = \beta \odot t_1 a t_2$ for some shorter path $\beta$ with $last(\beta ) =t_1$ and $a\in A$, $t_1, t_2 \in S$. By induction hypothesis, $l = RL _{\mathbf {y}(s)}^{}(\beta )\ge Safe _{\mathcal {M}}(t_1)$, from which it follows that $Safe _{\mathcal {M}}(t_1) < \infty$. Due to (1.), it follows that there exists at least one $x \in dom (\varSigma (t_1))$ such that $x \le l$. We select maximal *x* satisfying the inequality so that $a = \varSigma (t_1)(x)$. We have that $RL _{\mathbf {y}(s)}^{}(\alpha ) = RL _{l}^{}(t_1at_2)$ by definition and from (2.) it follows that $\bot \ne RL _{x}^{}(t_1at_2) \ge Safe (t_2)\ge 0$. All together, as $l \ge x$ we have that $RL _{\mathbf {y}(s)}^{}(\alpha ) \ge RL _{x}^{}(t_1at_2) \ge Safe (t_2)\ge 0$.    $\square$

Now we prove the part (a) of the main invariant. We show that throughout the execution of Algorithm 3, $\varSigma$ satisfies the assumptions of Lemma 7. Property (1.) is ensured by the initialization on line 3. The property (2.) holds upon first entry to the main loop by the definition of a safe action (Definition 6). Now assume that $\varSigma (s)(\mathbf {r}(s))$ is redefined on line 13, and let *a* be the action $\mathbf {a}(s)$.

We first handle the case when $s\not \in R$. Since *a* was selected on line 8, from the definition of  we have that there is $t \in Succ (s,a)$ such that after the loop iteration,

1 $$\begin{aligned} \mathbf {r}(s) = C(s,a) + \max \{\mathbf {r}_{ old }(t), Safe (t') \mid t\ne t'\in Succ (s,a)\} \ge C(s,a)+\max _{t' \in Succ (s,a)} Safe _{\mathcal {M}}(t'), \end{aligned}$$

the latter inequality following from Lemma 6. Satisfaction of property (2.) in *s* then follows immediately from the Eq. (1).

If $s \in R$, then (1) holds before the truncation on line 10, at which point $\mathbf {r}(s)< cap (\mathcal {M})$. Hence, $cap (\mathcal {M}) -C(s,a)\ge \max _{t \in Succ (s,a)} Safe _{\mathcal {M}}(t)$ as required by (2.). From Lemmas 6 and 7 it follows that $\varSigma ^{\mathbf {r}}$ is $Safe _{\mathcal {M}}(s)$-safe in every state *s*. This finishes the proof of part (a) of the invariant.

*Proving Part (b) of the Main Invariant.* Clearly, (b) holds after initialization. Now assume that an iteration of the main repeat loop was performed. Denote by $\pi _{ old }$ the strategy $\varSigma ^{\mathbf {r}_{ old }}$ and by $\pi$ the strategy $\varSigma ^{\mathbf {r}}$. Let *s* be any state such that $\mathbf {r}(s) \le cap (\mathcal {M})$. If $\mathbf {r}(s) = \mathbf {r}_{ old }(s)$, then we claim that (b) follows directly from the induction hypothesis: indeed, we have that there is an *s*-initiated $\pi _{ old }$-compatible path $\alpha$ ending in a target state s.t. the $\mathbf {r}_{ old }(s)$-initiated resource level along $\alpha$ never drops $\mathbf {r}_{ old }$, i.e. for each prefix $\beta$ of $\alpha$ it holds $RL _{\mathbf {r}_{ old }(s)}^{}(\beta )\ge \mathbf {r}_{ old }{( last(\beta ) )}$. But then $\beta$ is also $\pi$-compatible, since for each state *q*, $\varSigma (q)$ was only redefined for values smaller than $\mathbf {r}_{ old }(q)$.

The case when $$ \mathbf {r}(s) < \mathbf {r}_{ old }(s)$$ is treated similarly. As in the proof of part (a), denote by *a* the action $$ \mathbf {a}(s) $$ assigned on line 13. There must be a state $$ t \in Succ (s,a) $$ s.t. (1) holds before the truncation on line 10. In particular, for this *t* it holds $$ RL _{\mathbf {r}(s)}^{}(sat) \ge \mathbf {r}_{ old }(t)$$. By induction hypothesis, there is a *t*-initiated $$ \pi _{ old } $$-compatible path $$ \beta $$ ending in $$ T$$ satisfying the conditions in (b). We put $$ \alpha = s a t\odot \beta $$. Clearly $$\alpha $$ is *s*-initiated and reaches $$ T$$. Moreover, it is $$ \pi $$-compatible. To see this, note that $$\varSigma ^{\mathbf {r}}(s)(\mathbf {r}(s)) = a$$; moreover, the resource level after the first transition is $$ e(t) = RL _{\mathbf {r}(s)}^{}(sat) \ge \mathbf {r}_{ old }(t) $$, and due to the assumed properties of $$ \beta $$, the $$ \mathbf {r}_{ old }(t) $$-initiated resource level (with initial load *e*(*t*)) never decreases below $$ \mathbf {r}_{ old } $$ along $$\beta $$. Since $$ \varSigma $$ was only re-defined for values smaller than those given by the vector $$ \mathbf {r}_{ old } $$, $$ \pi $$ mimics $$ \pi _{ old } $$ along $$ \beta $$. Since $$ \mathbf {r} \le \mathbf {r}_{ old } $$, we have that along $$ \alpha $$, the $$ \mathbf {r}(s) $$-initiated resource level never decreases below $$ \mathbf {r} $$. This finishes the proof of part (b) of the invariant and thus also the proof of Theorem 7.   $$\square $$

## Büchi

This section proofs Theorem 1 which is the main theoretical result of the paper. The proof is broken down into the following steps.

1. We identify a largest set $R ^\prime \subseteq R$ of reload states such that from each $r\in R ^\prime$ we can reach $R ^\prime$ again (in at least one step) while consuming at most $cap$ resource units and restricting ourselves only to strategies that (i) avoid $R \setminus R ^\prime$ and (ii) guarantee positive reachability of $T$ in $\mathcal {M}( R ^\prime )$.
2. We show that $SafeB\ddot{u}chi _{\mathcal {M},T}= SafePR _{\mathcal {M}( R ^\prime ),T}$ and that the corresponding strategy (computed by Algorithm 3) is also $T$-Büchi $SafeB\ddot{u}chi _{\mathcal {M},T}(s)$-safe for each $s\in S$.

Algorithm 4 solves (1.) in a similar fashion as Algorithm 2 handled safety. In each iteration, we declare as non-reloading all states from which positive reachability of *T* and safety within $\mathcal {M}( Rel )$ cannot be guaranteed. This is repeated until we reach a fixed point. The number of iterations is clearly bounded by $| R |$.


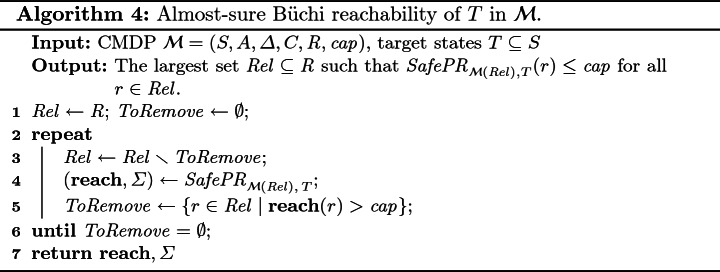


### Theorem 8

Let $\mathcal {M}= (S, A, \varDelta , C, R , cap )$ be a CMDP and $T\subseteq S$ be a target set. Moreover, let $R ^\prime$ be the contents of $Rel$ upon termination of Algorithm 4 for the input $\mathcal {M}$ and $T$. Finally let $\mathbf {r}$ and $\varSigma$ be the vector and the selector returned by Algorithm 3 for the input $\mathcal {M}$ and $T$. Then for every state *s*, the finite counter strategy $\sigma =\varSigma ^\mathbf {r}$ is *T*-Büchi $\mathbf {r}(s)$-safe in *s* in both $\mathcal {M}( R ^\prime )$ and $\mathcal {M}$. Moreover, the vector $\mathbf {r}$ is equal to $SafeB\ddot{u}chi _{\mathcal {M}, T}$.

### Proof

We first show that $\sigma$ is *T*-Büchi $\mathbf {r}(s)$-safe in $\mathcal {M}( R ^\prime )$ for all $s\in S$ with $\mathbf {r}(s) \le cap$. Clearly it is $\mathbf {r}(s)$-safe, so it remains to prove that *T* is visited infinitely often with probability 1. We know that upon every visit of a state $r\in R '$, $\sigma$ guarantees a future visit to *T* with positive probability. As a matter of fact, since $\sigma$ is a finite memory strategy, there is $\delta >0$ such that upon every visit of some $r\in R '$, the probability of a future visit to *T* is at least $\delta$. As $\mathcal {M}( R ^\prime )$ is decreasing, every *s*-initiated $\sigma$-compatible run must visit the set $R ^\prime$ infinitely many times. Hence, with probability 1 we reach *T* at least once. The argument can then be repeated from the first point of visit to *T* to show that with probability 1 ve visit *T* at least twice, three times, etc. *ad infinitum.* By the monotonicity of probability, $\mathbb {P}^{\sigma }_{\mathcal {M},s}(\mathsf {B\ddot{u}chi}_{T}) =1$.

It remains to show that $\mathbf {r}\le SafeB\ddot{u}chi _{\mathcal {M},T}$. Assume that there is a state $s\in S$ and a strategy $\sigma ^\prime$ such that $\sigma ^\prime$ is *d*-safe in *s* for some $d<\mathbf {r}(s) = SafePR _{\mathcal {M}( R '), T}(s)$. We show that this strategy is not *T*-Büchi *d*-safe in $\mathcal {M}$. If all $\sigma ^\prime$-compatible runs reach $T$, then there must be at least one history $\alpha$ produced by $\sigma ^\prime$ that visits $r\in R \setminus R ^\prime$ before reaching $T$ (otherwise $d\ge \mathbf {r}(s)$). Then either (a) $SafePR _{\mathcal {M}, T}(r) = \infty$, in which case any $\sigma '$-compatible extension of $\alpha$ avoids *T*; or (b) since $SafePR _{\mathcal {M}( R '), T}(r) > cap$, there must be an extension of $\alpha$ that visits, between the visit of *r* and $T$, another $r^\prime \in R \setminus R ^\prime$ such that $r^\prime \ne r$. We can then repeat the argument, eventually reaching the case (a) or running out of the resource, a contradiction with $\sigma ^\prime$ being *d*-safe.    $\square$

We can finally proceed to prove Theorem 1.

### Proof

*(of Theorem* 1*).* The theorem follows immediately from Theorem 8 since we can (a) compute $SafeB\ddot{u}chi _{\mathcal {M}, T}$ and the corresponding strategy $\sigma _T$ in polynomial time (see Theorem 7 and Algorithm 4); (b) we can easily check whether $d\ge SafeB\ddot{u}chi _{\mathcal {M}, T}(s)$, if yes, than $\sigma _T$ is the desired strategy $\sigma$; and (c) represent $\sigma _T$ in polynomial space as it is a finite counter strategy represented by a polynomial-size counter selector.    $\square$

## Implementation and Case Studies

We implemented the presented algorithms in Python and released it as an open-source tool called *FiMDP (Fuel in MDP)* available at https://github.com/xblahoud/FiMDP. The docker artifact is available at https://hub.docker.com/r/xblahoud/fimdp and can be run without installation via the Binder project [50]. We investigate the practical behavior of our algorithms using two case studies: (1) An autonomous electric vehicle (AEV) routing problem in the streets of Manhattan modeled using realistic traffic and electric car energy consumption data, and (2) a multi-agent grid world model inspired by the Mars Helicopter Scout [8] to be deployed from the planned Mars 2020 rover. The first scenario demonstrates the utility of our algorithm for solving real-world problems [59], while the second scenario studies the algorithm’s scalability limits.

The consumption-Büchi objective can be also solved by a naive approach that encodes the energy constraints in the state space of the MDP, and solves it using techniques for standard MDPs [33]. States of such an MDP are tuples (*s*, *e*) where *s* is a state of the input CMDP and *e* is the current level of energy. Naturally, all actions that would lead to states with $e < 0$ lead to a special sink state. The standard techniques rely on decomposition of the MDP into maximal end-components (MEC). We implemented the explicit encoding of CMDP into MDP, and the MEC-decomposition algorithm.

All computations presented in the following were performed on a PC with Intel Core i7-8700 3.20 GHz 12 core processor and a RAM of 16 GB running Ubuntu 18.04 LTS. All running times are means from at least 5 runs and the standard deviation was always below 5% among these runs.

**Fig. 2.**
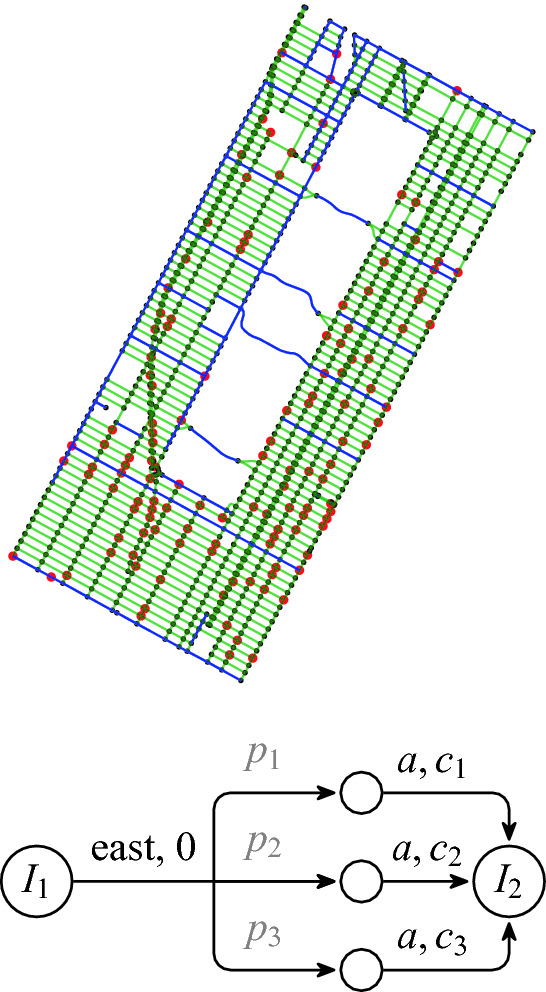
(Top:) Street network in the considered area. Charging stations are red, one way roads green, and two-way roads blue. (Bottom:) Transition from intersection $I_1$ to $I_2$ with stochastic consumption. The small circles are dummy states. (Color figure online)

### Electric Vehicle Routing

We consider the area in the middle of Manhattan, from 42nd to 116th Street, see Fig. 2. Street intersections and directions of feasible movement form the state and action spaces of the MDP. Intersections in the proximity of real-world fast charging stations [56] represent the set of reload states.

After the AEV picks a direction, it reaches the next intersection in that direction deterministically with a stochastic energy consumption. We base our model of consumption on distributions of vehicle travel times from the area [55] and conversion of velocity and travel times to energy consumption [52]. We discretize the consumption distribution into three possible values ($c_1, c_2, c_3$) reached with corresponding probabilities ($p_1, p_2, p_3$). We then model the transition from one intersection ($I_1$) to another ($I_2$) using additional dummy states as explained in Fig. 2.

**Fig. 3.**
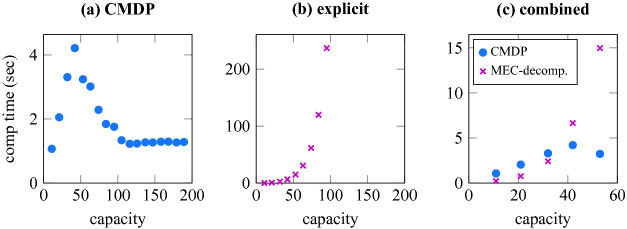
Mean computation times for a fixed target set of size 100 and varying capacity: **(a) CMDP** – computating Büchi objective via CMDP, **(b) explicit** – computating MEC decomposition of the explicit MDP, **(c) combined** – **(a)** and **(b)** combined for small capacity values.

The corresponding CMDP has 7378 states and 8473 actions. For a fixed set of 100 randomly selected target states, Fig. 3 shows influence of requested capacity on running times for **(a)** strategy for Büchi objective using CMDP (our approach), and **(b)** MEC-decomposition for the corresponding explicit MDP. With constant number of states, our algorithm runs reasonably fast for all capacities and the running time stabilizes for $$ cap > 95$$; this is not the case for the explicit approach where the number of states keeps growing (52747 for $$ cap =95$$) as well as the running time. The decomposition to MECs is slightly faster than solving Büchi using CMDP for the small capacities (Fig. 3 (c)), but MECs decomposition is only a part of the solution and running the full algorithm for Büchi would most likely diminish this advantage.

### Multi-agent Grid World

**Fig. 4.**
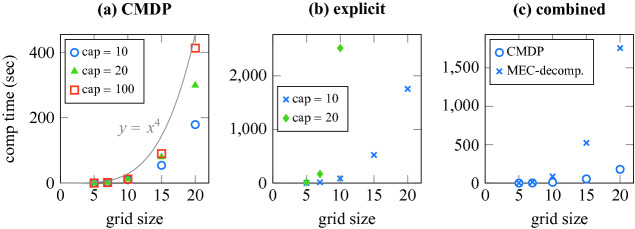
Mean computation times for varying grid sizes and of size capacities: **(a) CMDP** – computating Büchi objective via CMDP, the gray line shows the corresponding growth in the number of states on separate scale, **(b) explicit** – computating MEC decomposition of the explicit MDP, **(c) combined** – combined computation time for a capacity of 10.

We use multi-agent grid world to generate CMDP with huge number of states to study the scalability limits of the proposed algorithms. We model the rover and the helicopter of the Mars 2020 mission with the following realistic considerations: the rover enjoys infinite energy while the helicopter is restricted by batteries recharged at the rover. These two vehicle jointly operate on a mission where the helicopter reaches areas inaccessible to the rover. The outcomes of the helicopter’s actions are deterministic while those of the rover—influenced by terrain dynamics—are stochastic. For a grid world of size *n*, this system can be naturally modeled as a CMDP with $n^4$ states. Figure 4 shows the running times of the Büchi objective for growing grid sizes and capacities in CMDP. We observe that the increase in the computational time of CMDP follows the growth in the number of states roughly linearly, and our implementation deals with an MDP with $1.6\times 10^5$ states in no more than seven minutes. The figure also shows the running time for the MEC decomposition of the corresponding explicit MDP when the capacity is 10 and, for certain smaller, computationally feasible grid sizes, when the capacity is 20.

## Conclusion and Future Work

We presented a first study of consumption Markov decision processes (CMDPs) with qualitative $\omega$-regular objectives. We developed and implemented a polynomial-time algorithm for CMDPs with an objective of probability-1 satisfaction of a given Büchi condition. Possible directions for the future work are extensions to quantitative analysis (e.g. minimizing the expected resource consumption), stochastic games, or partially observable setting.

## Footnotes

1 In some literature, the level is required to stay positive as opposed to non-negative, but this is only a matter of definition: both approaches are equivalent.

2 In our model, this is equivalent to requiring that with probability 1, the resource level never drops below 0.
